# Toward strong, transparent and science-based dietary guidance: lessons learned from the Mediterranean Diet guideline development

**DOI:** 10.3389/fnut.2025.1708486

**Published:** 2025-12-05

**Authors:** Laura Rossi, Vincenza Gianfredi, Antonella Lezo, Stefania Maggi, Daniele Nucci, Graziano Onder, Marco Silano, Ersilia Troiano, Massimo Volpe, Michela Zanetti, Nicola Veronese

**Affiliations:** 1 Department of Food Safety, Nutrition, and Veterinary Public Health, National Institute of Health, Rome, Italy; 2 Department of Cardiac Thoracic Vascular Sciences and Public Health, University of Padua, Padua, Italy; 3 Dietetic and Clinical Nutrition Unit, Regina Margherita-S.Anna Hospital, Città della Salute e della Scienza, Turin, Italy; 4 CNR Aging Branch, Institute of Neuroscience, Padua, Italy; 5 Struttura Semplice Dipartimentale Igiene Alimenti e Nutrizione, Dipartimento di Igiene e Prevenzione Sanitaria, Agenzia di Tutela della Salute (ATS) Brescia, Brescia, Italy; 6 PhD National Program in One Health Approaches to Infectious Diseases and Life Science Research, Department of Public Health, Experimental and Forensic Medicine, University of Pavia, Pavia, Italy; 7 Università Cattolica del Sacro Cuore, Rome, Italy; 8 Fondazione Policlinico Gemelli IRCCS, Rome, Italy; 9 Department of Cardiovascular and Endocrine-Metabolic Diseases and Aging, National Institute of Health, Rome, Italy; 10 Department of Socio-Educational Services, Municipality Rome III, Rome, Italy; 11 Scientific Association for Food, Nutrition, and Dietetics (ASAND), Rome, Italy; 12 IRCCS San Raffaele Roma, Rome, Italy; 13 Università di Roma Sapienza, Rome, Italy; 14 UCO Geriatria, Dipartimento di Scienze Mediche, Chirurgiche e della Salute, Università degli Studi di Trieste, Trieste, Italy; 15 Faculty of Medicine, Saint Camillus University, Rome, Italy; 16 Unità Locale Socio-Sanitaria 3, Department of Primary Care, Venice, Italy

**Keywords:** Mediterranean Diet, dietary guidelines, consensus document, multidisciplinary commission, public health, Italy

## Abstract

The Mediterranean Diet Guidelines (MDGs) represent a structured, evidence-informed effort to redefine and promote the Mediterranean Diet (MD) as a tool for health promotion and disease prevention. This work originated from a rigorous methodological process that integrates systematic reviews, expert consensus, and NUTRIGRADE-based evaluations to generate actionable recommendations. The updated definition of the MD extends beyond its nutritional aspects to embrace key sociocultural dimensions, such as culinary traditions, conviviality, and sustainability. This reconceptualization positions the MD as a holistic lifestyle model rather than a restrictive dietary pattern. It also reflects current scientific and public health priorities by explicitly excluding alcohol consumption, including red wine, from its core recommendations. This decision acknowledges growing evidence that even moderate alcohol intake increases the risk of cancer and cardiovascular disease. The MDGs addressed a broad spectrum of health outcomes. They highlighted varying degrees of positive association between adherence to MD and reduced incidence or mortality across different conditions. Adherence to the MD showed strong protective effects against cardiovascular and metabolic diseases. On the other hand, its association with oncological, neurocognitive, musculoskeletal, and autoimmune conditions appears to be positive, though supported by weaker evidence. Specific recommendations are tailored for different life stages and target groups, including children, pregnant women, older adults, and individuals with chronic conditions. Dissemination and implementation strategies emphasize integration into clinical care, schools, public policies, and digital health platforms. The MDGs provide a scalable and adaptable framework for national and regional adoption, promoting guideline harmonization. MD is thereby recognized as a pivotal public health tool in the face of global nutritional transitions and rising non-communicable diseases.

## 1 Introduction

The Mediterranean Diet (MD) is linked to a set of skills, knowledge, practices, and traditions ranging from cultivation to the processing, preparation, and consumption of food and defines a unique lifestyle recognized as a common cultural heritage of Mediterranean communities (1). MD is widely recognized as a model of healthy eating, with higher adherence consistently associated with significant improvements in health and nutritional status (2). Numerous studies have linked greater adherence to MD with reduced all-cause mortality and lower incidence of chronic conditions, including cardiovascular disease, type 2 diabetes, various cancers, and neurodegenerative disorders (3). MD has also been recognized as a sustainable diet having a low environmental impact (4).

### 1.1 Mediterranean Diet: from epidemiology to recommendations

Transformations in the global food system have led to significant shifts in dietary patterns from traditional to more convenient, processed, and less nutritious food choices, with negative health consequences like overweight, obesity, diabetes, elevated blood pressure, and hyperlipidemia, all metabolic risk factors for Non-Communicable Diseases (NCDs) (5). This trend is also impacting Mediterranean countries, where a growing Westernization of food consumption patterns has been observed (6). Italy follows this trend, with a decline in adherence to the MD well-documented in several studies (7, 8), despite variations in the indices used for assessment. Notably, a cross-sectional survey on the Adherence to the MD in Italy (ARIANNA), based on a sample of 3,732 Italian adults, showed that only 5% of respondents achieved high adherence to the MD (9). Moreover, recent findings from a study conducted on a sample of 2,869 adults found that only 13% of the population reported high adherence to the MD (10).

The MD is widely referenced in national dietary guidelines across countries in the Mediterranean basin. For instance, the Italian Food-Based Dietary Guidelines (FBDGs) explicitly state that their recommendations are inspired by and aligned with the principles of the MD (11). Similarly, the Turkish dietary guidelines endorse the MD as a model for healthy eating (12). In Spain, the most recent national guidelines (2022) affirm the alignment of their dietary recommendations with the MD (13). The Lebanese guidelines include several recommendations that explicitly reference the traditional Lebanese diet, which shares many core characteristics with the MD (14). Likewise, the Maltese guidelines, titled *“Healthy Eating the Mediterranean Way!”*, make direct reference not only to the MD but also to the associated lifestyle practices (15).

### 1.2 Translating evidence into dietary guidance for public health

The elaboration of FBDGs is grounded in scientific evidence, even if their scope and complexity vary considerably across nations. Some countries rely primarily on systematic literature reviews and expert opinions, while others incorporate quantitative modeling based on national food consumption data in the formulation of their guidelines (16). Although dietary guidelines continue to be developed using non-harmonized methodologies, considerable progress has been achieved. The Position Paper by the Academy of Nutrition Sciences (17) highlights this advancement by reviewing the scientific evidence and methodological developments in nutritional epidemiology that support dietary recommendations for preventing non-communicable diseases. It underscores the importance of transparent, evidence-based processes for updating recommendations, the need for rigorous criteria to assess experimental data, and improved methods for measuring dietary intake. On the other hand, the development of guidelines in clinical settings tends to be more standardized than the elaboration of nutritional dietary guidelines. For example, the World Health Organization (WHO) guidelines system ensures that recommendations are based on the best available evidence and aligned with internationally recognized standards.^1^ In Italy the National Institute of Health (Istituto Superiore di Sanità–ISS) coordinates the National Guidelines System (SNLG), which guarantees methodological quality and consistency in the formulation of evidence-based recommendations. Recognizing the importance of effectively translating research into public health guidance and bridging the gap between clinical and population-level dietary recommendations, the Italian Society for Artificial Nutrition and Metabolism (SINPE), the Mediterranean Diet Foundation (FDM), and the Italian Society for Cardiovascular Prevention (SIPREC), in collaboration with the Italian National Institute of Health (ISS) and with the collaboration of other 20 scientific societies including physicians, dieticians, nurses and stakeholders have promoted the elaboration of the Mediterranean Diet Guidelines (MDGs) in accordance with the SNLG procedures. The development of MDGs was undertaken to provide a standardized, evidence-based framework that extends beyond existing FBDGs, addressing clinical, methodological, and sustainability dimensions of the MD through the formal application of the SNLG process. In other terms, the MDGs were developed to complement, rather than replace, existing FBDGs by offering a scientifically robust, multidisciplinary, and harmonized framework that strengthens the application of the MD across public health and healthcare systems. Unlike the previous work by Rossi et al. (11), which focused on the methodological framework and development process of the Italian FBDGs, the present study specifically details the evidence-based development of the MDGs, employing a distinct methodological approach and scope aimed at translating those principles into an operational and applied model for evidence-based dietary guidance.

### 1.3 Objectives, research questions, and hypothesis

The primary objective of this paper is to describe the methodological process behind the development of the MDGs, with particular emphasis on the strengths and limitations of the approaches and methodologies applied. This is especially relevant as it marks the first time that the SNLG procedure has been adopted for the development of nutritional recommendations in Italy. Specifically, the paper aims to: (i) explore opportunities to enhance the development of evidence-based dietary recommendations using the MDGs as a case study; (ii) propose future directions for strengthening the scientific rigor, transparency, and methodological consistency of nutrition guidance; and (iii) outline strategies for the dissemination and implementation of the MDGs across diverse settings.

The research questions underlying this work are: (i) what lessons can be drawn from the development of the MDGs to inform the creation of more robust, evidence-based, and harmonized dietary guidelines? (ii) in what ways do the MDGs contribute to bridging the gap between clinical and population-level dietary guidance, particularly in the context of sustainable and culturally rooted diets? (iii) What are the barriers or leverages for an effective dissemination and implementation of the MDGs in various clinical, public health, and policy settings?

The translational, methodological, and policy-oriented nature of this work lays is grounded in the following hypotheses: (i) the adoption of standardized, evidence-based methodologies in the development of the MDGs reinforces the scientific validity, transparency, and relevance of dietary recommendations, ensuring their usability across both clinical and public health domains; (ii) the MDGs serve as a model for international harmonization in the formulation of dietary guidelines, particularly within the Mediterranean region, offering a replicable framework that integrates cultural specificity with methodological rigor; (iii) the structured and participatory development process underpinning the MDGs strengthens their potential for effective dissemination and implementation across varied population contexts. This enhances their capacity to influence dietary behaviors and supports transitions toward healthier and more sustainable eating patterns on a scale.

## 2 Assessment of Mediterranean Diet guidelines options and implications

The MDGs were developed to establish, based on the best available scientific evidence, the conceptual, scientific, and procedural foundations for applying the principle of the MD across a wide range of clinical contexts and pathological conditions. These include its use in prevention, treatment, care, and rehabilitation. Clinical and research recommendations were developed on the following topics: (i) how MD is defined in scientific literature and the tools and methodologies to assess adherence to MD and its impact on health outcomes; and (ii) the effectiveness of the MD in reducing the risk of or positively influencing the progression of adverse health outcomes associated with various diseases. Each research question addressed in the Guideline, along with its corresponding recommendations, was developed separately according to the principles of primary and tertiary prevention. Primary prevention focuses on assessing the effectiveness of the MD in reducing the risk of developing chronic diseases. In contrast, tertiary prevention explores how adherence to the MD can help mitigate adverse outcomes in individuals with pre-existing conditions and support recovery or the maintenance of quality of life in patients with chronic or advanced diseases (18). The Guideline also provides a clear and widely accepted definition of the MD, examines its fundamental characteristics, and includes an evaluation of its relative costs and benefits.

### 2.1 The MDGs development process

The guideline development process applied the GRADE methodology (19), with specific adaptations for the field of nutrition such as the NUTRIGRADE approach (20) which emphasize assessing the quality of the available evidence and, specifically in the context of guidelines development, the strength of recommendations. These procedures adhere to international standards that ensure a transparent, systematic, and reproducible process for evidence evaluation and recommendation formulation, consistent with accepted principles for trustworthy guidelines (21). The initial process involves the formation of a multidisciplinary team of experts, which formulates the specific questions using the so-called PICO framework (Patients, Interventions or Exposure, Comparisons, Outcomes) (22). The development of the Italian MDGs was initiated by three Scientific Societies (SINPE, FDM, and SIPREC) in partnership with the ISS and carried out in alignment with the procedures established by SNLG. Given the considerable heterogeneity of contexts in which the MD is applied, the guideline development process was designed to include methodological and procedural standardization. This approach aims to ensure that the resulting recommendations are aligned with the healthcare and social realities in which the MD is implemented. To achieve this, the promoters of the guideline chose to involve, from the earliest stages, a broad range of professionals from the health and social sectors, as well as representatives from civil society.

#### 2.1.1 Organizational framework of the MDGs

**FIGURE 1:**
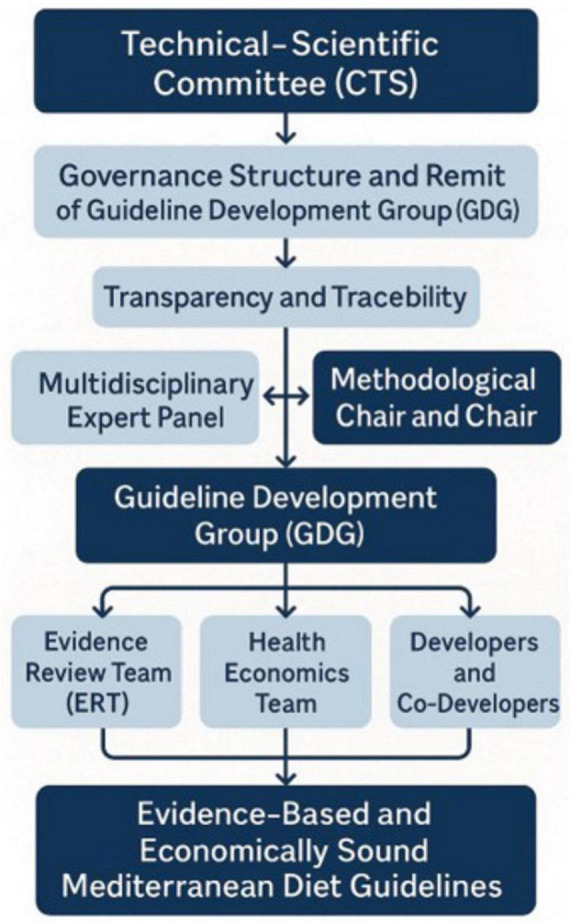
Mediterranean Diet guidelines development structure.

The MDGs organizational framework is shown in Figure 1 and included a Technical–Scientific Committee (CTS) that oversaw the project’s governance, defining the structure and remit of the Guideline Development Group (GDG), selecting panel members to guarantee the inclusion of key stakeholders and patient advocates, and coordinating all activities to maintain transparency and traceability. CTS issued an open call to scientific societies, professional organizations, consumer associations, and health policymakers; each responding body appointed an expert to the Multidisciplinary Expert Panel, which participated in every phase of the guideline, from scoping and PICO question formulation to recommendations’ drafting. A Methodological Chair and Co-Chair worked alongside the GDG to refine objectives, oversee literature reviews, and guide recommendation development, ensuring continuous engagement of the expert panel. The operational work was supported by specialized teams: the Evidence Review Team (ERT) conducted, synthesized, and presented systematic reviews for each PICO question; the Health Economics Team provided cost-effectiveness analyses and integrated economic considerations into recommendation formulation; and the Developers and Co-Developers coordinated activity flow, applied the GRADE and NUTRIGRADE methodologies, and safeguarded the transparency and traceability of all guideline development procedures. The formulation of PICO questions was guided by predefined criteria established under the SNLG, emphasizing clinical and public health relevance, feasibility, and evidence of availability. Each question was developed through a structured consensus process involving the GDG and validated by the CTS before official registration and approval by the SNLG, ensuring methodological standards for guideline development. In accordance with SNLG procedures, all members of the GDG and ERT declared potential Conflicts of Interest (COIs) at the outset and were monitored throughout the process. Financial and non-financial, personal, and institutional interests relevant to the guideline’s objectives were disclosed and assessed by the CTS based on their nature, relevance, timing, and potential impact on recommendations. When necessary, management measures were applied, such as temporary exclusion from specific decision-making activities. All COIs were collectively reviewed and classified following the SNLG methodological manual. Only minimal or non-relevant COIs were identified, none of which affected the scientific neutrality of the recommendations. Full declarations are archived with the Guideline Secretariat and disclosed in the final document of the MDGs. The Chair and Co-Chairs oversaw this process to ensure transparency and prevent bias in evidence evaluation and recommendation formulation. No external funding was received for the preparation of MDGs.

### 2.2 The role of the national guidelines system

The Italian MDGs were elaborated under the institutional umbrella of the National Guidelines System (Sistema Nazionale Linee Guida—SNLG) of the ISS. The SNLG is grounded in the best available evidence and is tailored to address Italy’s specific health needs, based on clinical, economic, and social relevance criteria. The SNLG serves as the institutional access point for clinical and public health guidelines developed for the National Health Service, offering guidance to decision-makers, healthcare professionals, and patients.^2^ The SNLG addresses a wide range of topics related to clinical and care best practices, including areas where official guidelines have yet to be developed. This approach aims to provide reliable references for healthcare professionals, public health practitioners, and SNLG users, supporting clinical practice, public health initiatives, and, in some cases, broader health policy decisions. In practice, the SNLG system interprets guideline development in line with the definition provided by United States National Academy of Medicine, where clinical practice guidelines are recommendations aimed at optimizing patient care. These guidelines are grounded in systematic reviews of the available evidence and provide a clear assessment of the benefits and risks associated with alternative care options (23). The elaboration of the MDGs represents a landmark development in the history of the SNLG: for the first time, its methodological framework was applied to the domain of nutrition and dietary guidance. This innovation required adapting established procedures, such as the GRADE and NUTRIGRADE methodologies, to evaluate the quality of nutritional evidence and the strength of dietary recommendations. By applying these rigorous standards to a complex, multidimensional topic such as the MD, the MDGs have set a new precedent for the integration of nutritional science into formal public health guideline systems. This process not only enhances the credibility and usability of the MDGs but also provides a replicable model for the development of future nutrition-related guidance within national and international public health frameworks.

### 2.3 The definition of MD

The definition of the MD has progressively evolved from a traditional set of dietary practices into a scientifically grounded and multidimensional model for promoting health and sustainability (24). While originally rooted in the historical food patterns of populations inhabiting the Mediterranean basin, contemporary understandings of the MD encompass not only nutritional content but also sociocultural, environmental, and lifestyle dimensions (25).

The MDGs developed under the SNLG include a clear and consensual definition of the MD. This definition is the product of a structured process aimed at synthesizing the most recurrent and validated elements across available literature. An introductory question was formulated to elaborate both the conceptual and operational definition of the MD. It aimed to identify the shared “core” components across existing definitions and to describe the tools used to assess adherence, along with their modes of application.

Since Ancel Keys’ foundational work in the 1950s, which first framed the MD as a coherent dietary model, the scientific understanding of the MD has significantly deepened. Subsequent decades have yielded numerous refinements, including updated food pyramids and diverse adherence scoring systems (26). However, the comparative analysis of the different definitions revealed no substantial discrepancies in the core food components that characterize the MD.

From a conceptual standpoint, the MD is increasingly recognized not simply as a dietary regimen, based on seasonality, locally and traditional relevant food, and practices/recipes aimed at reducing food waste but as a sustainable and culturally embedded lifestyle. This broader vision incorporates conscious food choices, regular physical activity, sufficient sleep and rest, conviviality during meals, culinary skills, and social connectedness. The values of hospitality, neighborliness, intercultural dialogue, and respect for cultural diversity further enrich this holistic perspective (1).

Thus, the MD is better understood today as a comprehensive lifestyle, sometimes referred to as the “Mediterranean way of life” (27). Updating the traditional 1960s model of the MD is essential—not only to reflect the evolving scientific evidence base, but also to accommodate changes in lifestyles and social norms both within and beyond the Mediterranean region (28).

#### 2.3.1 Defining the Mediterranean Diet in practice

The operational definition of the Mediterranean Diet, as developed through expert consensus, emphasizes a dietary pattern predominantly based on plant-derived foods. These include generous daily intakes of vegetables, fruits, legumes, whole grains, and nuts, with extra virgin olive oil as the principal source of dietary fat. The model advocates moderate consumption of fish, seafood, poultry, eggs, milk, and dairy products, while recommending limited intake of red and processed meats. Occasional consumption of sweets and a minimal presence of ultra-processed foods are also consistent with this pattern. As a largely plant-based and minimally processed dietary model, the MD promotes the consumption of seasonal, locally sourced, and culturally relevant foods. Importantly, its scope extends beyond nutrient composition to encompass social and cultural dimensions—including the value of communal meals, the safeguarding of local food systems, and the preservation of intergenerational culinary traditions that strengthen social cohesion and cultural continuity (29). To assess adherence, the MDGs identified and described 15 validated instruments, such as the Mediterranean Diet Score (MDS) (30), MEDAS (31), and MEDI-LITE (32), that evaluate core aspects of MD compliance. These tools allow for standardized assessment across research and clinical settings and help track the implementation of MD-based interventions in public health and healthcare systems.

Two particularly innovative aspects in the definition of MD, as articulated in the MDGs, deserve emphasis. First, MD is conceptualized not merely as a set of nutritional prescriptions but as a holistic lifestyle framework that integrates cultural and social dimensions of eating. This broader perspective assigns equal importance to the practice of shared meals, the safeguarding of local and traditional food systems, and the intergenerational transmission of culinary knowledge and practices. These sociocultural components are regarded as equally fundamental as the inclusion of specific food groups or traditional ingredients in defining the MD pattern. This emphasis is particularly relevant within the framework of the MDGs, which are primarily guided by public health principles and aim to promote sustainable, culturally rooted dietary practices not only at the population level but also in the health care system. The second innovative aspect of the MDGs is the explicit exclusion of alcohol, including red wine, from the definition. Although wine was traditionally associated with Mediterranean eating habits, emerging scientific evidence now links even moderate alcohol consumption to increased risks of cancer (33) and cardiovascular disease (34). MDGs position on alcohol aligns with public health guidance, including that of the WHO, which states that no level of alcohol consumption is safe (35). Despite the cultural significance of wine in Mediterranean societies and its inclusion as a positive element in several indices of adherence to the MD, the MDGs prioritize health and sustainability by adopting an evidence-based approach that excludes alcohol, particularly given the absence of robust randomized trials (34, 36). This position is further supported by the updated Italian Society of Human Nutrition Mediterranean Diet Pyramid (37), reflecting a shift toward a more protective and health-conscious interpretation of the Mediterranean lifestyle that integrates tradition with modern scientific understanding (38).

### 2.4 The main recommendations

The MDGs examinate 10 PICO questions covering the mortality level (Q1) and specific health domains, cardiovascular, metabolic, oncologic, neurological, musculoskeletal, disability and frailty in the elderly, and autoimmune diseases (Q2–Q8). These were categorized into two major levels of prevention: primary (preventing disease onset—Q2A–Q8A) and tertiary (improving outcomes in individuals with existing disease—Q2B–Q8B). Additionally, the effect of MD on maternal-child health was assessed (Q9). Finally, economic impact (Q10A) and environmental sustainability were also analyzed (Q10B). The MDGs issued a total of 111 recommendations: 83 operational recommendations, based on a systematic review of evidence using the NUTRIGRADE methodology, and 28 research recommendations formulated to address existing gaps, advance knowledge and inform future research priorities. Recommendations were classified according to two dimensions: the direction of effect (beneficial or not beneficial) and the confidence in the supporting evidence, following the principles of the GRADE and NUTRIGRADE frameworks. The terms “strong” and “weak” reflect an adaptation of the NUTRIGRADE scoring system for clearer policy and communication purposes. While the grading of evidence quality followed the NUTRIGRADE thresholds for strength and certainty, the qualitative labels were simplified to enhance interpretability for non-academic audiences. Differences between observational and interventional data were addressed through a structured grading process. Observational studies, which constitute most of the available evidence, were evaluated for consistency, strength of association, and biological plausibility, while interventional studies were prioritized for causal inference when available. When both types of evidence were present, the confidence level reflected an integrated assessment of study design, methodological quality, and coherence of findings. Quantitative synthesis relied on pooled Relative Risks (RRs) from existing meta-analyses, ensuring standardized and comparable estimates of the association between MD adherence and key health outcomes. This approach guaranteed transparency and scientific rigor while acknowledging the inherent limitations of nutritional research. When evidence from Mediterranean and non-Mediterranean populations differed, recommendations were harmonized through expert consensus, emphasizing the consistency of biological mechanisms and the transferability of dietary patterns over geographic context. Where stronger evidence derived from Mediterranean cohorts and weaker or heterogeneous results were observed elsewhere, the direction of the recommendation was maintained but its strength was adjusted accordingly, ensuring evidence-based yet context-sensitive guidance.

#### 2.4.1 All-cause mortality

The first MDGs PICO question concerned the impact of MD on overall mortality in the general population. A total of 57 cohort and retrospective studies, involving 1,833,267 participants and an average follow-up of 13 years (range: 2–60 years), were included in the analysis. These studies collectively recorded 346,034 deaths, representing approximately 19% of the study population. The pooled RR estimate was 0.96 (95% IC: 0.95–0.97) for each one-point increase in MD adherence score, indicating a modest but consistent protective association. Although no RTCs were available, the quality of the observational evidence was rated as moderate using the NUTRIGRADE methodology. This rating reflects a favorable risk of bias profile, good precision, and acceptable reproducibility, albeit with some heterogeneity in MD definitions and follow-up durations. Sensitivity analyses further confirmed the consistency of the protective effect across age groups (adults vs. elderly) and sexes (men vs. women), with no statistically significant interaction observed. These findings demonstrated the role of MD as a primary prevention strategy for reducing all-cause mortality. Despite the observational nature of the evidence, the balance of desirable and undesirable effects, acceptability, feasibility, and potential to promote equity and sustainability all support the adoption of the MD as a population-wide public health strategy (29).

**FIGURE 2:**
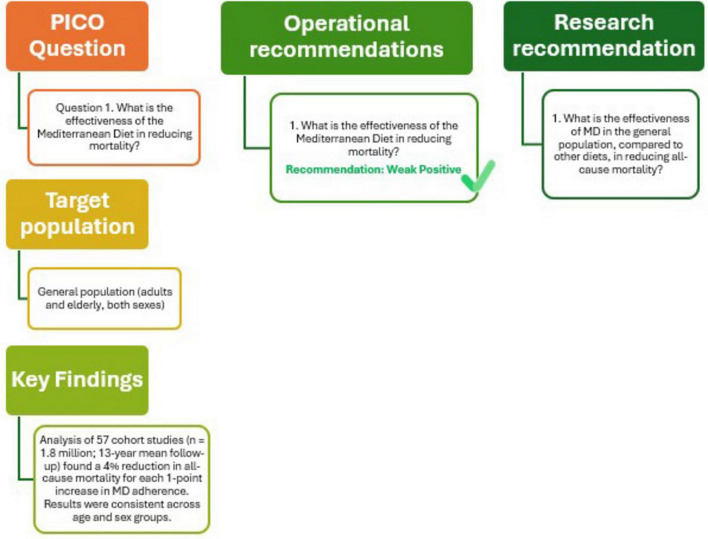
Mediterranean Diet and all-cause mortality.

The outcomes of this analysis, presented in Figure 2 (Q1), support the recommendation that adherence to the MD is an effective strategy to reduce all-cause mortality in the general population. The confidence in the supporting evidence is moderate positive. Future research should aim to further evaluate and compare the effectiveness of MD against other dietary patterns in lowering all-cause mortality in the general population.

#### 2.4.2 Cardiovascular health

##### 2.4.2.1 Primary prevention

PICO Q2A explored the relationship between MD and primary prevention of cardiovascular diseases. One of the most influential studies, the PREDIMED, a large multicenter RTC conducted in Spain, demonstrated that a MD enriched with extra virgin olive oil or nuts significantly reduces the risk of major cardiovascular events in individuals at high-risk of CVD. Specifically, participants in the MD groups experienced a 30–40% relative risk reduction in combined outcomes of myocardial infarction, stroke, and cardiovascular death compared to those on a low-fat control diet (39). Further analyses of PREDIMED and additional cohort studies have shown that MD adherence is associated with a lower incidence of stroke, atrial fibrillation, and peripheral artery disease, with beneficial effects attributed to anti-inflammatory, antioxidant, and endothelial-protective mechanisms (40). The evidence for cardiovascular protection is strengthened by the availability of randomized trial data, as well as by consistent findings in observational studies. These studies collectively highlight that greater adherence to the MD results in meaningful improvements in lipid profiles, blood pressure control, and markers of endothelial function. In addition, the inclusion of olive oil, nuts, fruits, vegetables, and whole grains, key components of the MD, supports a diet pattern rich in monounsaturated fats, fiber, and polyphenols, all of which contribute to cardiovascular benefits (41). Overall, the certainty of evidence supporting cardiovascular protection from the MD is rated as high to moderate, with strong consistency across studies and populations, and a favorable balance of desirable effects, minimal risk, and broad acceptability. These findings demonstrated that MD is an effective tool in both primary and secondary prevention of cardiovascular disease (29).

**FIGURE 3:**
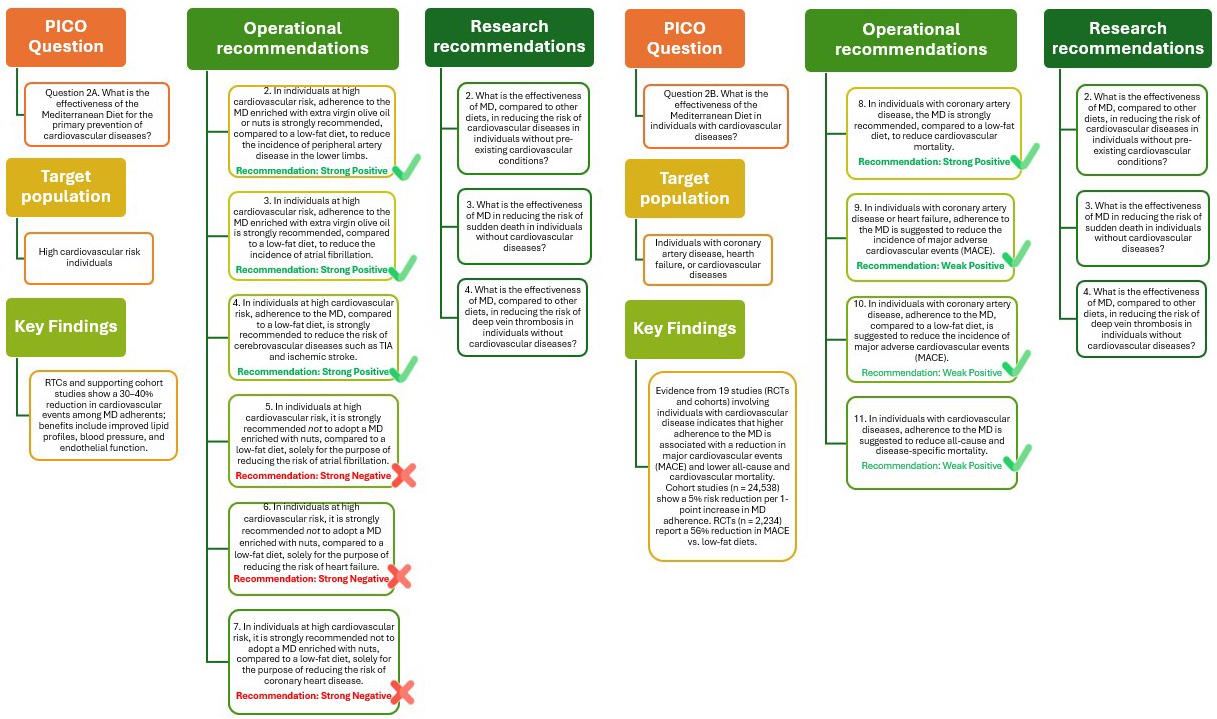
Mediterranean Diet and primary (Q2A) and tertiary (Q2B) prevention of cardiovascular diseases.

The outcome of this analysis is illustrated in Figure 3 (Q2A) and supports the recommendation that in high-risk and general populations, adherence to the MD is strongly recommended for the prevention of cardiovascular diseases, including stroke and coronary heart disease. The quality of the evidence of the recommendation is strongly positive. Future research should focus on the effectiveness of the Mediterranean Diet, compared to other diets, in reducing cardiovascular risk, sudden death, and deep vein thrombosis in individuals without cardiovascular diseases.

##### 2.4.2.2 Improving outcomes in individuals with cardiovascular disease

The effectiveness of the MD in individuals already affected by cardiovascular diseases has been evaluated through a body of evidence comprising Randomized Controlled Trials (RCTs) and observational cohort studies. A total of 19 studies were included in the analysis, covering a range of cardiovascular conditions such as coronary artery disease, myocardial infarction, and heart failure. Outcomes assessed included Major Adverse Cardiovascular Events (MACE), all-cause and cardiovascular mortality, blood pressure, metabolic markers, and body composition. For the outcome of MACE, six cohort studies including 24,538 participants with cardiovascular disease showed a statistically significant protective association with higher MD adherence. The pooled RR was 0.95 (95% IC: 0.93–0.97) per one-point increase in MD adherence, with a moderate certainty of evidence. Complementing this, three RCTs (*n* = 2,234) comparing the MD to low-fat or standard diets demonstrated a larger protective effect, with an RR of 0.44 (95% IC: 0.20–0.94), albeit with low certainty due to methodological limitations and sample size constraints. Regarding mortality outcomes, 12 cohort studies (*n* = 91,747) documented a significant inverse association between MD adherence and both all-cause and cardiovascular mortality (RR: 0.96, 95% IC: 0.95–0.98). Evidence for other clinical outcomes, such as blood pressure, body composition, and lipid and glucose profiles, remains limited. One RCT found no statistically significant effect of the MD on systolic or diastolic blood pressure compared to a low-fat diet. Similar null effects were reported for cholesterol (both low- and high-density lipoproteins), triglycerides, and fasting glucose. However, a small trial did report an increase in lean body mass percentage (+ 3.4%, 95% IC: 2.59–4.21%) in the MD group, suggesting potential benefits on body composition.

Despite the relatively low number of RCTs, the evidence indicates that adherence to the MD can contribute to reducing cardiovascular events and mortality in individuals with existing Cardiovascular Disease (CVD). The demonstrated benefits, minimal risk of adverse effects, high acceptability, strong feasibility, and alignment with sustainability principles warrant the inclusion of the Mediterranean Diet as an effective component of tertiary prevention strategies (29). The results of this analysis, summarized in Figure 3 (Q2B), provide consistent evidence supporting the beneficial role of the Mediterranean Diet (MD) in individuals with CVD. In particular, adherence to the MD is strongly recommended for people with coronary artery disease, as it has been shown to significantly reduce cardiovascular mortality compared to low-fat diets. For individuals with coronary artery disease or heart failure, following the MD is suggested to lower the incidence of MACE, although the supporting evidence is of moderate strength. Similarly, in patients with existing CVD, adherence to the MD is associated with reductions in both all-cause and cardiovascular-specific mortality, supported by evidence of moderate to low certainty. Overall, these findings reinforce the role of MD as a key component in the secondary prevention and management of cardiovascular diseases. However, further research is needed to clarify its effectiveness in specific clinical outcomes, such as hospitalization rates, blood pressure regulation, lipid profile improvement (particularly LDL cholesterol), prevention of diabetes onset, and enhancement of metabolic parameters.

#### 2.4.3 Oncological diseases

##### 2.4.3.1 Primary prevention

The positive effects of MD on oncological disease risk have been increasingly examined in scientific literature over the past decade (42). Although its health benefits are most strongly established in cardiovascular and metabolic domains, an expanding body of evidence indicates that adherence to the MD may also play a meaningful role in the primary prevention of cancer (43). This protective effect appears most consistent for colorectal, breast (particularly postmenopausal), gastric, and prostate cancers, where large cohort studies and meta-analyses report relative risk reductions ranging from 10 to 20% for individuals in the highest MD adherence categories compared to those with the lowest (44).

The biological plausibility of this association is underpinned by several key features of the MD: a high intake of fiber, antioxidants, and anti-inflammatory compounds derived from vegetables, fruits, legumes, whole grains, olive oil, and nuts; a low consumption of red and processed meats; and a preference for minimally processed, seasonal, and locally sourced foods (45). These elements are thought to contribute to reduced chronic inflammation, improved insulin sensitivity, and protection against DNA damage and oxidative stress—all mechanisms implicated in carcinogenesis (46).

Despite these findings, it is important to acknowledge that the current body of evidence is predominantly observational, with a paucity of RCTs directly assessing cancer outcomes as primary endpoints. Moreover, heterogeneity in MD definitions and scoring tools limits comparability across studies. As such, the certainty of the evidence is considered moderate based on frameworks such as NUTRIGRADE and GRADE (29).

**FIGURE 4:**
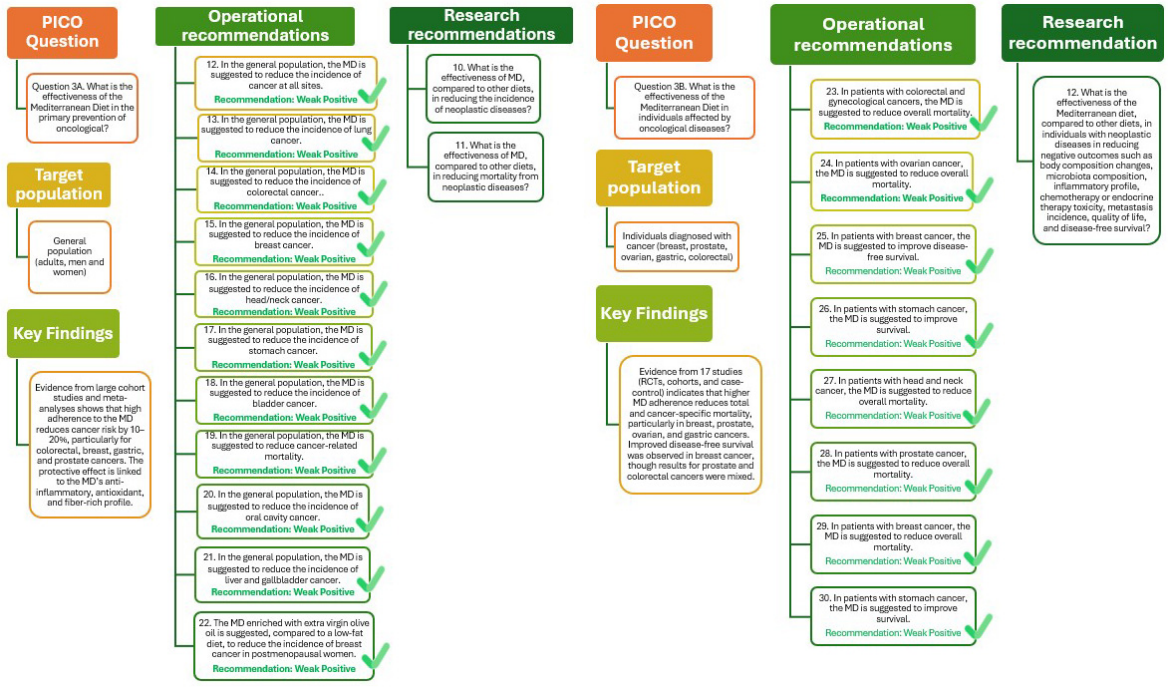
Mediterranean Diet and primary (Q3A) and tertiary (Q3B) prevention of oncological diseases.

The analysis presented in Figure 4 (Q3A) supports the recommendation that adherence to MD should be promoted within the general population as an effective preventive measure to reduce cancer risk. The overall quality of the supporting evidence is considered moderately positive, reflecting consistent findings across multiple studies, despite some variability in study design and population characteristics. Further research is needed to better define the effectiveness of the MD compared with other dietary patterns in reducing both the incidence and mortality of neoplastic diseases, in order to strengthen causal inference and inform future dietary guidelines for cancer prevention.

##### 2.4.3.2 Improving outcomes in individuals with oncological diseases

The PICO question Q3B assessed the potential efficacy of the MD in individuals with oncological diseases. A total of 17 studies, comprising RTCs, cohort, and case-control studies, were included in the evidence analysis. These studies, although heterogeneous in terms of cancer type, adherence scores, and follow-up durations, provide preliminary insights into the potential benefits of MD across various oncological outcomes.

The evidence suggests that higher adherence to the MD is associated with modest reductions in total mortality among individuals affected by cancer, particularly in patients with breast, prostate, ovarian, and gastric cancers. For example, in women with breast cancer, pooled results from five cohort studies involving over 13,000 participants showed a reduction in both overall and cancer-specific mortality (RR = 0.97; 95% IC: 0.96–0.98). Similar protective trends were noted in prostate cancer (RR = 0.97; 95% IC: 0.95–0.99), ovarian cancer (RR = 0.68; 95% CI: 0.56–0.87), and stomach cancer (RR = 0.50; 95% IC: 0.45–0.55). Disease-free survival also appeared to improve breast cancer (RR = 0.39; 95% IC: 0.15–0.72), although findings in prostate cancer were less conclusive. In contrast, evidence for colorectal cancer was mixed. While one cohort study found no significant association with MD adherence (RR = 0.99), a case-control study suggested a stronger protective effect (RR = 0.62; 95% IC: 0.39–0.96). The only RCT identified (47) reported low-certainty evidence on body composition and quality of life in prostate cancer patients, showing a slight improvement in quality of life but some reduction in lean mass.

The quality of evidence was rated as moderate for most mortality outcomes, although limited by study heterogeneity, varying definitions of MD, and a small number of RCTs. Despite these constraints, the findings indicate that adherence to the MD may offer complementary benefits when integrated into supportive care for cancer patients. Further high-quality, large-scale studies are needed to confirm these associations and to clarify the potential advantages of the MD across different tumor types and treatment phases (29).

As shown in Figure 4 (Q3B), these results support the recommendation that adherence to MD can be beneficial for patients with oncological diseases, contributing to improved survival and overall clinical outcomes. The certainty of evidence supporting this recommendation is considered weakly positive. Future research should evaluate the effectiveness of the MD compared to other diets in mitigating adverse outcomes, including changes in body composition and microbiota, inflammation, treatment-related toxicity, metastasis, quality of life, and disease-free survival.

#### 2.4.4 Neurological diseases and mental health

##### 2.4.4.1 Primary prevention

The MDGs literature review examining the role of MD in the primary prevention of neurological diseases included 44 studies, both observational and interventional, evaluating the association between MD adherence and various outcomes, such as Alzheimer’s disease (AD), dementia, depression, anxiety, mild cognitive impairment (MCI), and Parkinson’s disease. Six cohort and case-control studies involving over 36,000 participants reported a significant association between higher MD adherence and reduced risk of Alzheimer’ disease (RR = 0.92, 95% IC: 0.87–0.98), with a moderate level of evidence. Similar results were observed for MCI incidence (RR = 0.93, 95% IC: 0.88–0.98) and for depression incidence (RR = 0.96, 95% IC: 0.94–0.97), supported by studies involving nearly 85,000 individuals. The MD also showed promise in reducing the prevalence of anxiety (OR = 0.89, 95% IC: 0.82–0.97) and depression (OR = 0.91, 95% IC: 0.87–0.95), as well as the incidence of Parkinson’s disease (RR = 0.90, 95% IC: 0.83–0.97). Despite differences in dietary assessment methods and follow-up durations, the consistency of protective associations across different conditions and populations strengthens the evidence for the preventive role of MD in neurological health. The overall certainty of the evidence was rated as moderate, supporting positive but cautious recommendations given the predominance of observational studies (29).

**FIGURE 5:**
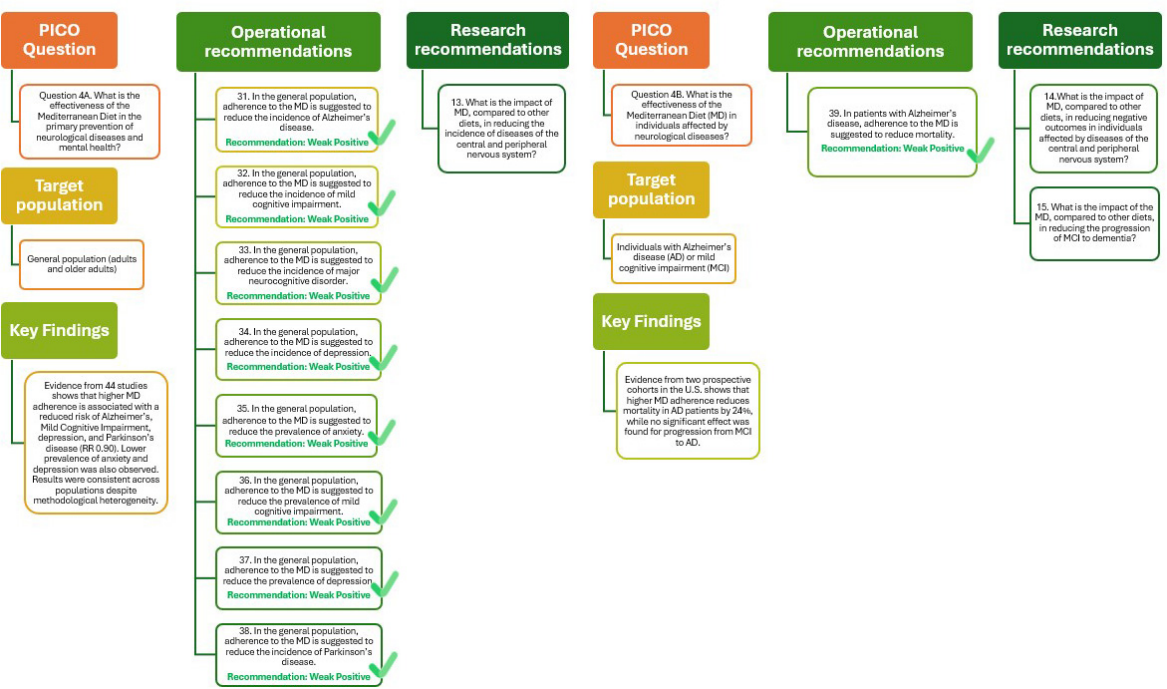
Mediterranean Diet and primary (Q4A) and tertiary (Q4B) prevention of neurological diseases and mental health.

As shown in Figure 5 (Q4A) these findings support the recommendation that, in the general population, adherence to the MD helps reduce the incidence of Alzheimer’s disease, MCI, depression, anxiety, and Parkinson’s disease. The strength of this recommendation is considered weakly positive reflecting the need for more robust experimental data. Future research should further assess the impact of the MD compared with other dietary patterns in preventing diseases of the central and peripheral nervous systems, thereby reinforcing its role in public health strategies for cognitive and mental well-being.

##### 2.4.4.2 Improving outcomes in individuals with neurological diseases and mental health

The impact of adherence to the MD on neurological outcomes has been explored in a limited number of observational studies, with a focus on elderly populations without initial frailty or disability. Two prospective cohort studies conducted in the United States (48, 49) evaluated the effect of MD on AD and MCI. The first study followed 192 individuals with AD over a 4.4-year period and reported a statistically significant reduction in mortality among those with higher MD adherence. Specifically, each one-point increase in the MD adherence score was associated with a 24% lower risk of death (HR = 0.76; 95% CI: 0.65–0.89). This association was supported by moderate-quality evidence, as assessed by the NUTRIGRADE methodology (48).

In contrast, the second study included 482 participants with MCI and evaluated the progression to Alzheimer’s disease over 4.3 years. The results did not reach statistical significance (HR = 0.89; 95% CI: 0.78–1.02), and the quality of evidence was rated as low. These findings indicate that adherence to the MD may help in reduce mortality in individuals with AD, although current evidence is insufficient to confirm its role in preventing the progression from MCI to dementia. The limited number of studies, absence of RTCs, and heterogeneity in MD definitions underscore the need for further high-quality research to confirm these preliminary results and to identify subgroups that may benefit most (49).

As shown in Figure 5 (Q4B) the analysis supports the recommendation that adherence to the MD may be beneficial for reducing mortality in people with AD, with the quality of evidence rated as weakly positive. Future studies should explore the impact of MD compared with other dietary patterns in reducing adverse outcomes in neurological diseases and slowing the progression of MCI to dementia.

#### 2.4.5 Metabolic diseases

##### 2.4.5.1 Primary prevention

To evaluate the effectiveness of MD in preventing metabolic diseases a total of 60 studies were identified, encompassing over one million participants. Adherence to the MD was consistently associated with a lower risk of developing type 2 diabetes, overweight, and obesity. Notably, 18 cohort studies reported a 4% risk reduction in type 2 diabetes incidence for each one-point increase in MD adherence score (RR = 0.96; 95% CI: 0.95–0.97), supported by moderate-certainty evidence. The PREDIMED trial further confirmed a protective effect of MD enriched with extra virgin olive oil, showing a 20% reduced risk compared to a low-fat diet (RR = 0.80; 95% CI: 0.70–0.92).

Similarly, MD adherence was linked to a reduced incidence and prevalence of overweight and obesity, both in adults and in developmental ages (children and adolescents). The association with other conditions such as hypercholesterolemia, hypertriglyceridemia, and metabolic syndrome was less conclusive, with weaker or non-significant associations and a lower certainty of evidence. While overall findings suggest a protective role of MD against several metabolic disorders, heterogeneity in diet definitions, follow-up durations, and outcome measures limit the generalizability of results (29).

**FIGURE 6:**
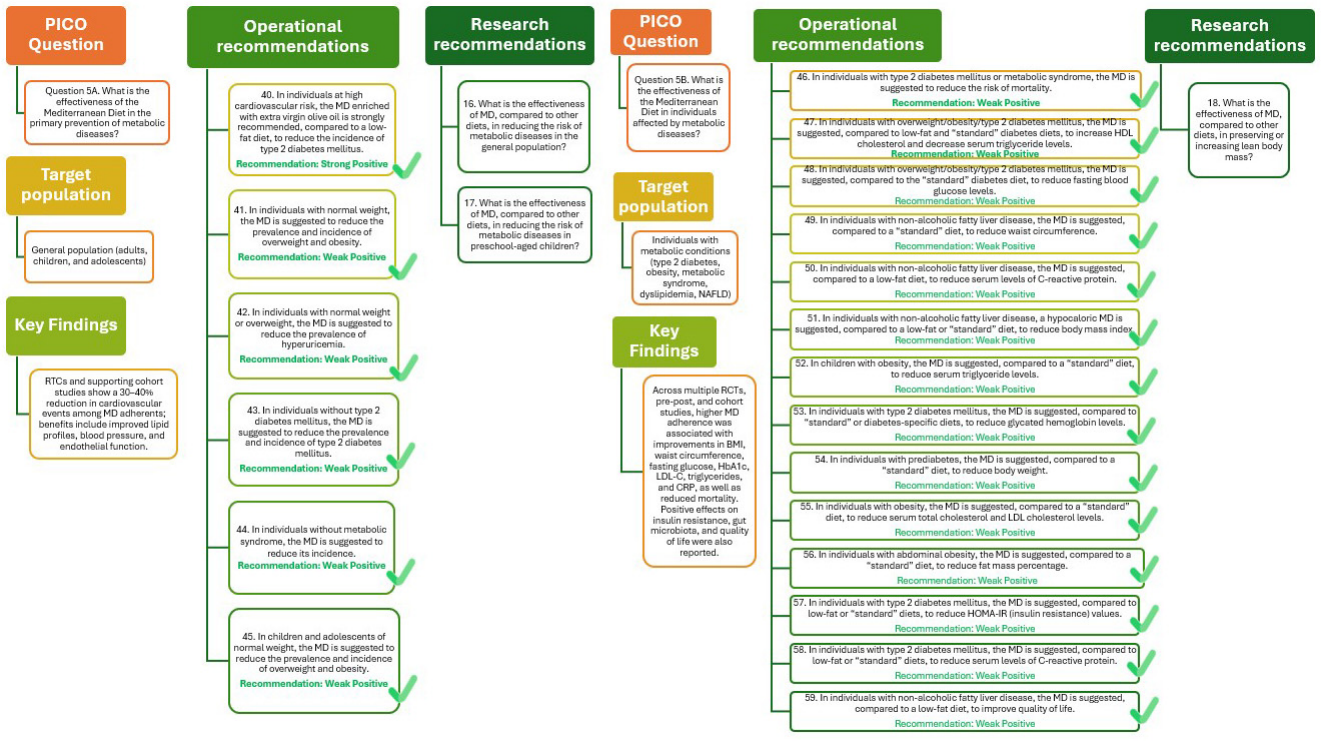
Mediterranean Diet and primary (Q5A) and tertiary (Q5B) prevention of metabolic diseases.

The analysis presented in Figure 6 (Q5A) supports the recommendations that adherence to MD plays an important role in the prevention of metabolic diseases. In individuals at high cardiovascular risk, a MD enriched with extra virgin olive oil is strongly recommended to reduce the incidence of type 2 diabetes, supported by high-quality evidence. Among individuals without diabetes, including younger populations, adherence to the MD is suggested to lower the risk and prevalence of type 2 diabetes, overweight, obesity, metabolic syndrome, and hyperuricemia, although the certainty of evidence remains limited. Further research should clarify the effectiveness of the MD compared with other dietary patterns in preventing metabolic diseases, particularly in preschool-aged children and other underrepresented groups.

##### 2.4.5.2 Improving outcomes in individuals with metabolic diseases

A wide range of studies, comprising RTCs, pre-post interventions, and observational cohorts, was assessed to evaluate the effectiveness of MD in managing metabolic conditions such as type 2 diabetes, metabolic syndrome, dyslipidemia, non-alcoholic fatty liver disease (NAFLD), and obesity. The available data show that higher adherence to the MD is associated with improvements in numerous clinically relevant outcomes, including reductions in body mass index, waist circumference, fat mass, fasting glucose, glycated hemoglobin, Low-Density Lipoprotein Cholesterol, triglycerides, C-reactive protein, and mortality. Furthermore, MD may positively influence insulin resistance and gut microbiota composition and improve quality of life in selected populations. While the overall certainty of evidence is moderate for most outcomes, heterogeneity in study design, diversity in comparator diets, and the frequent use of pre-post designs without controls limit the strength of conclusions. Nonetheless, the MD appears to be a promising dietary strategy in the management of metabolic diseases (29).

The analysis presented in Figure 6 (Q5B) indicate that, among individuals with metabolic diseases, such as type 2 diabetes, metabolic syndrome, and NAFLD, adherence to the MD may contribute to improved cardiometabolic outcomes and enhanced quality of life. Although the available evidence supports a positive association, its overall certainty is limited, largely due to variability in study designs and comparators. Further research is needed to better define the effectiveness of the MD compared with other dietary approaches, particularly regarding its potential to preserve or increase lean body mass and support long-term metabolic health.

#### 2.4.6 Musculoskeletal disorders

##### 2.4.6.1 Primary prevention

The potential of MD to prevent musculoskeletal conditions has been explored through 15 observational studies involving diverse populations, including children, adults, and older individuals. The studies examined outcomes such as osteoporosis, sarcopenia, and fracture incidence. Regarding osteoporosis, one cross-sectional study in children and young adults found no significant association with MD adherence (OR = 1.00; 95% CI: 0.89–1.12), while another study in adults suggested a possible protective effect (OR = 0.77; 95% CI: 0.60–1.00), although both findings were based on low-certainty evidence.

More robust findings emerged for fracture prevention. Eight cohort and case-control studies, involving over 655,000 participants and recording 6,746 events, indicated a small but statistically significant reduction in fracture risk associated with higher MD adherence (RR = 0.97; 95% CI: 0.94–0.99). The evidence quality for this outcome was rated as moderate. In contrast, evidence on sarcopenia was less conclusive: two cohort studies and four cross-sectional studies found no significant associations for either incidence (OR = 0.89; 95% CI: 0.76–1.05) or prevalence (OR = 0.94; 95% CI: 0.85–1.05), both supported by low-certainty evidence.

Despite the lack of RTCs and variability in MD definitions, follow-up durations, and measurement tools, these results suggest that MD may play a modest role in reducing fracture risk and possibly osteoporosis in adults. These findings highlight the need for further high-quality research to better define MD’s role in musculoskeletal health and to identify subpopulations who may benefit most (29).

**FIGURE 7:**
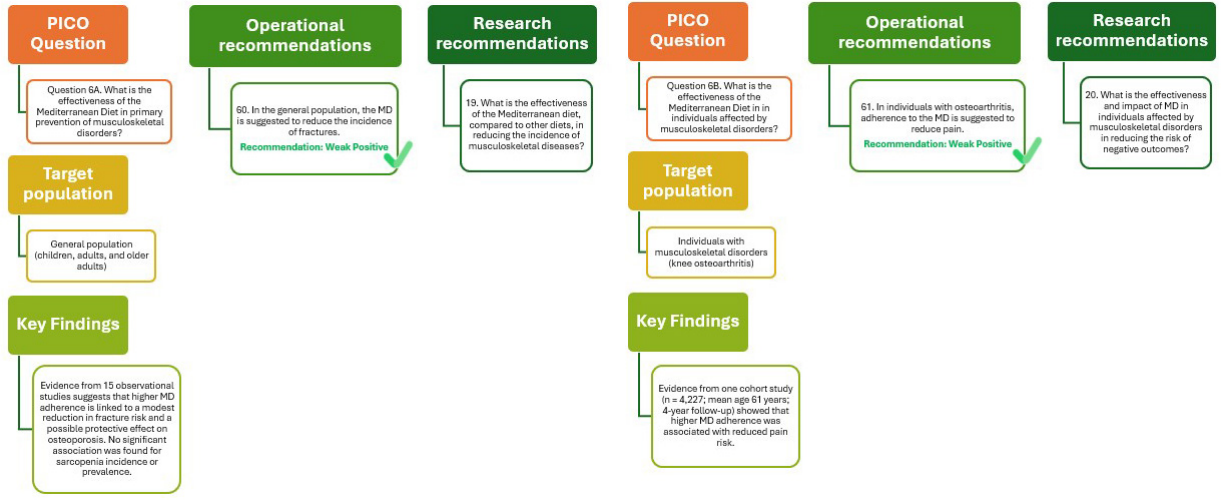
Mediterranean Diet and primary (Q6A) and tertiary (Q6B) prevention of musculoskeletal disorders.

As shown in Figure 7 (Q6A), the analysis supports the recommendation that adherence to the MD may help reduce fracture incidence in the general population, though the quality of evidence remains weakly positive. Further studies are warranted to strengthen the understanding of the effectiveness of the MD compared with other diets in preventing musculoskeletal diseases.

##### 2.4.6.2 Improving outcomes in individuals with musculoskeletal disorders

The potential role of MD in alleviating symptoms and improving outcomes in individuals already affected by musculoskeletal disorders has been minimally explored. Only one observational cohort study met inclusion criteria, evaluating the relationship between MD adherence and pain in patients with knee osteoarthritis (50). This study included 4,227 participants with a mean age of 61.1 years and followed them over a period of 4 years. The analysis revealed a small but statistically significant association between higher MD adherence and reduced pain risk (HR = 0.98; 95% CI: 0.97–0.998 for each one-point increase in MD adherence score). The quality of evidence for this outcome was rated as moderate using the NUTRIGRADE system, supported by methodological rigor and population relevance.

The analysis presented in Figure 7 (Q6B) suggests that in individuals with osteoarthritis, adherence to the MD may help in reduce pain and improve overall well-being. Although the evidence base remains limited, primarily derived from a single observational study (29), the findings point toward a potential role for the MD in managing musculoskeletal disorders. The quality of evidence supporting this recommendation is considered weakly positive, highlighting the need for further well-designed studies (e.g., RTCs) to confirm these effects. Future research should focus on clarifying the effectiveness and broader impact of MD, compared with other dietary patterns, in reducing adverse outcomes in individuals affected by musculoskeletal diseases.

#### 2.4.7 Frailty and disability in older adults

##### 2.4.7.1 Primary prevention

A total of 19 studies, comprising prospective cohorts and cross-sectional analyses, examined the relationship between MD adherence and the onset or prevalence of frailty and disability in individuals aged 65 years and older, who were free from these conditions at baseline. The overall evidence indicates a modest but consistent protective effect of high MD adherence on these outcomes. Specifically, nine cohort studies involving 94,072 older adults demonstrated a reduced risk of incident frailty (OR = 0.95; 95% CI: 0.93–0.97 per one-point increase in MD adherence), with moderate certainty of evidence. Similarly, six cross-sectional studies showed an association with lower frailty prevalence (OR = 0.94; 95% CI: 0.90–0.98), although with lower certainty. Regarding disability, two studies found a statistically significant inverse association with disability prevalence (OR = 0.98; 95% CI: 0.97–0.98), supported by moderate-certainty evidence. However, evidence from three cohort studies on disability incidence was inconclusive (OR = 0.96; 95% CI: 0.89–1.03), and the overall certainty was rated low. Although RTCs are lacking and some heterogeneity exists in how both the MD and frailty/disability are defined, the available evidence suggests that adherence to the MD may help delay functional decline and support healthy aging. Its adoption MD among older adults represents a low-risk, culturally appropriate approach to maintaining autonomy and quality of life (29).

**FIGURE 8:**
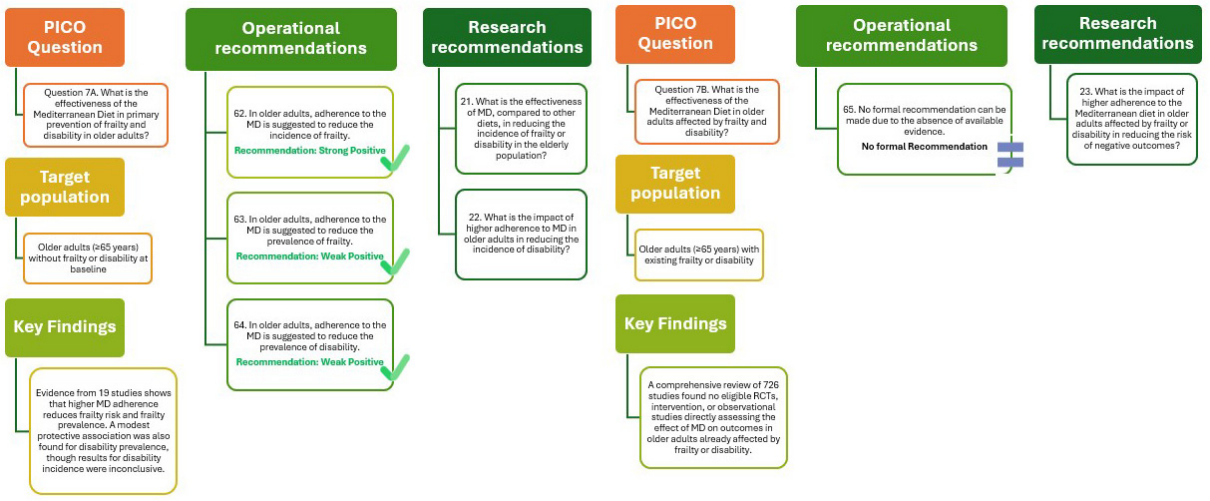
Mediterranean Diet and primary (Q7A) and tertiary (Q7B) prevention of frailty and disability in older adults.

As shown in Figure 8 (Q7A), the analysis supports the recommendations that in older adults, adherence to the MD is associated with lower incidence and prevalence of frailty, and lower prevalence of disability, although the strength of evidence is weakly positive. Future studies should further explore the effectiveness of the MD compared with other diets in reducing frailty and disability and assess how higher adherence may influence the preservation of functional independence in aging populations.

##### 2.4.7.2 Improving outcomes in older adults with frailty and disability

The effectiveness of the MD in improving health outcomes among older adults (≥ 65 years) already affected by frailty or disability remains currently unexplored in scientific literature. A comprehensive review of 726 studies did not identify any RTCs, pre-post intervention studies, or observational studies addressing the impact of MD adherence on reducing adverse outcomes in this specific population group (29).

The outcome of this analysis is illustrated in Figure 8 (Q7B) and supports the finding that no formal recommendation can be made due to the absence of available evidence. Future studies should further explore the impact of higher adherence to MD in older adults with frailty or disability, particularly regarding its potential to reduce the risk of adverse outcomes.

#### 2.4.8 Autoimmune diseases

##### 2.4.8.1 Primary prevention

The relationship between adherence to the MD and the primary prevention of autoimmune diseases has been explored through nine observational studies involving large, general population cohorts. These studies investigated the incidence or prevalence of several autoimmune conditions, including rheumatoid arthritis, systemic lupus erythematosus, Crohn’s disease, ulcerative colitis, multiple sclerosis, and Sjögren’s syndrome. Overall, the available evidence suggests a potential protective effect of high MD adherence against the development of multiple sclerosis (51, 52) and Sjögren’s syndrome (53). However, for other autoimmune conditions such as rheumatoid arthritis, lupus, and inflammatory bowel diseases, no significant associations were observed. The certainty of evidence for all outcomes was rated as low due to heterogeneity in MD definitions, variability in follow-up durations, and the absence of RCTs. Nevertheless, the consistency of results observed for certain outcomes and the absence of significant adverse effects support the potential of MD as a preventive strategy against autoimmune diseases, particularly in conditions where early modulation of inflammatory pathways may offer clinical benefits (29).

**FIGURE 9:**
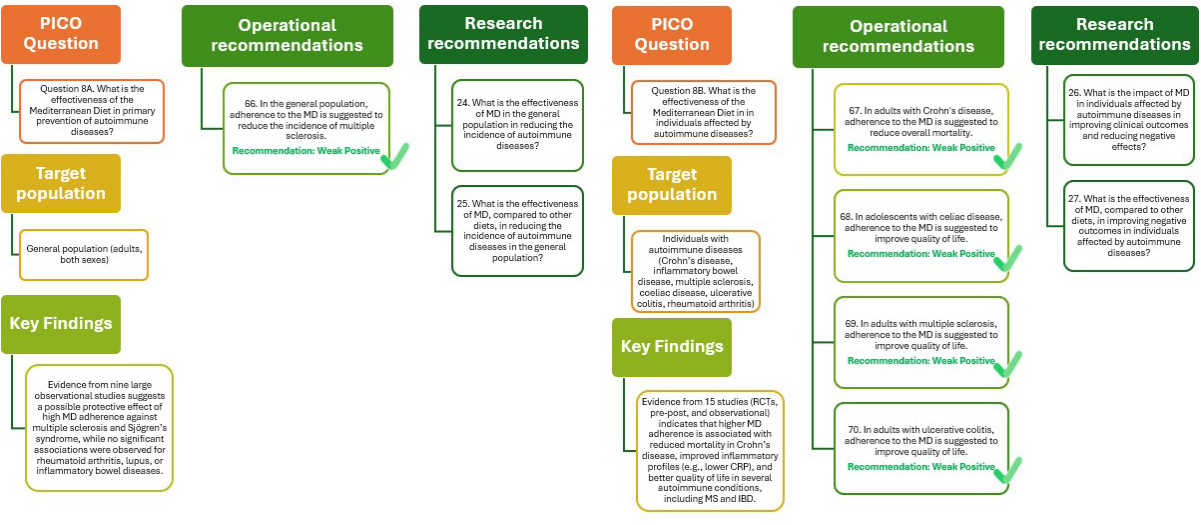
Mediterranean Diet and primary (Q8A) and tertiary (Q8B) prevention of autoimmune diseases.

As illustrated in Figure 9 (Q8A), the analysis supports the recommendation that adherence to the MD may help reduce the incidence of multiple sclerosis in the general population, although the quality of evidence remains positive. Future research should further evaluate the effectiveness of the MD compared with other dietary patterns, with a focus on long-term outcomes and specific autoimmune conditions.

##### 2.4.8.2 Improving outcomes in individuals with autoimmune diseases

The effectiveness of the MD in individuals with autoimmune diseases has been evaluated across 15 studies, including RCTs, pre-post intervention designs, and observational research. The outcomes assessed ranged from body composition, inflammatory markers, and gut microbiota to overall mortality and quality of life. In patients with Crohn’s disease, MD adherence was significantly associated with reduced all-cause mortality (HR = 0.5, 95% CI: 0.29–0.87), with moderate certainty of evidence. For inflammatory bowel disease more broadly, a lower mortality risk and improved inflammatory profiles (e.g., reduced C-reactive protein levels) were also reported, though with low certainty. Regarding quality of life, positive associations were observed in individuals with multiple sclerosis, coeliac disease, ulcerative colitis, and rheumatoid arthritis, particularly among adolescents and those with higher MD adherence. Although these findings indicate promising benefits, the overall certainty of the evidence remains low due to the limited number of high-quality RCTs, variability in study designs, and heterogeneity in outcome measurement. Nevertheless, these results suggest the MD could be a valuable approach in the management of autoimmune conditions (29).

The analysis presented in Figure 9 (Q8B) supports the recommendations that that adherence to the MD may offer meaningful benefits for individuals with autoimmune diseases. In adults with Crohn’s disease, the MD is suggested to reduce overall mortality, while in those with multiple sclerosis, ulcerative colitis, and in adolescents with coeliac disease, it appears to improve quality of life. Although these findings are encouraging, the quality of the evidence remains weakly positive, reflecting the limited number of studies and variability in design. Further research should clarify the impact of higher adherence to the MD on reducing adverse outcomes in vulnerable populations, including older adults with frailty or disability.

#### 2.4.9 Benefits of MD in pregnancy

The potential benefits of the MD during pregnancy have been evaluated in 33 studies, including RCTs, cohort studies, and case-control designs, encompassing over 120,000 pregnant women. These investigations explored a wide range of maternal and neonatal outcomes. High adherence to the MD, especially when enriched with extra virgin olive oil and nuts, was consistently associated with a reduced risk of key adverse outcomes, particularly gestational diabetes, low birth weight, and preterm birth. In RTCs involving more than 2,600 women, the MD significantly reduced the risk of gestational diabetes compared to standard or low-fat diets (RR = 0.74, 95% CI: 0.63–0.88), with high certainty. A similar protective effect was observed for low birth weight (RR = 0.61, 95% CI: 0.42–0.88) and for preterm birth (RR = 0.45, 95% CI: 0.05–0.84), both with moderate to high certainty. Additional benefits included lower incidence of infections and reduced neonatal respiratory distress. While the effects on outcomes such as fetal growth alterations, pre-eclampsia, and neonatal death were modest, the consistency across diverse settings and the low risk of bias enhance the reliability of these findings. Nevertheless, variability in MD definitions and follow-up durations highlight the need for further high-quality research to address underexplored outcomes and refine dietary recommendations. Overall, the evidence warrants MD as an effective and safe dietary approach to improve maternal and fetal health (29).

**FIGURE 10:**
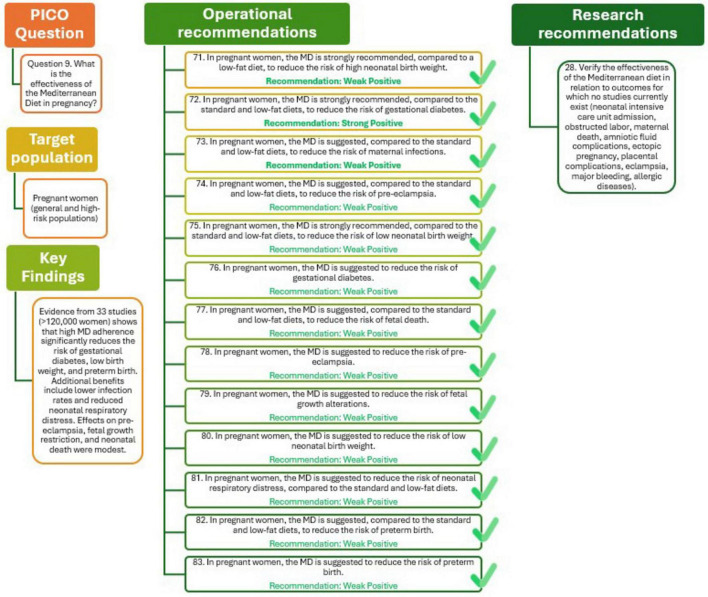
Mediterranean Diet in pregnancy (Q9).

The analysis presented in Figure 10 (Q9) highlights the beneficial role of MD during pregnancy. Evidence strongly supports that, compared with low-fat or standard diets, adherence to the MD reduces the risk of high and low birth weight as well as gestational diabetes, with high-quality evidence underpinning these outcomes. The MD is also suggested to lower the risk of preterm birth, pre-eclampsia, fetal growth restriction, neonatal infections, respiratory distress at birth, and fetal mortality, although the certainty of this evidence is weaker due to study variability.

Further research should assess the effectiveness of the MD in relation to outcomes not yet studied, such as neonatal intensive care admission, obstructed labor, maternal death, amniotic fluid or placental complications, eclampsia, major bleeding, and allergic diseases, to provide a more comprehensive understanding of its impact on maternal and neonatal health.

#### 2.4.10 Economic impact and sustainability of the Mediterranean Diet

A total of 61 studies were included to assess the economic and environmental implications of MD, reflecting its potential as a sustainable public health strategy. Among these, 23 studies focused on the economic dimension. Evaluations ranged from cost-effectiveness analyses and cost-utility models to broader cost-of-illness simulations. Overall, high adherence to the MD was associated with reduced direct healthcare costs, particularly in relation to cardiovascular disease prevention, and improvements in quality-adjusted life years (QALY). Cost-effectiveness was more pronounced in long-term models, with several studies (54, 55) reporting favorable incremental cost-effectiveness ratios under various scenarios. While most studies adopted healthcare systems or societal perspectives, variations in cost structures, healthcare settings, and population dietary habits limited generalizability. Despite methodological heterogeneity and context-specific assumptions, the MD consistently demonstrated good value for money in primary and secondary prevention strategies. Additionally, interventions promoting the MD appeared cost-saving or cost-neutral even under conservative assumptions. However, key limitations included short time horizons, lack of randomized trial data in some cases, and limited applicability to specific health systems (29).

The reported analysis supports the following recommendations:

• In public health strategies, MD should be considered cost-effective and economically sustainable for reducing healthcare burdens, particularly for chronic diseases such as cardiovascular conditions and type 2 diabetes.
• Policymakers are encouraged to promote MD adherence through fiscal and educational interventions, considering both long-term health outcomes and healthcare savings.

The quality of evidence is positive, conditional on context and implementation model.

## 3 Actionable recommendations

According to the outcomes of MDGs, MD has demonstrated effectiveness on all-cause mortality, across a wide spectrum of health outcomes, and in terms of sustainability and economic impact. To translate these scientific findings into meaningful public health impact, it is essential to develop actionable strategies that support the dissemination, adoption, and sustained implementation of the MD principles. Key recommendations for operationalizing the evidence, identifying appropriate settings for intervention, defining priority target groups, engaging relevant stakeholders, and establishing effective dissemination and implementation mechanisms were proposed. The goal is to foster an enabling environment where adherence to the MD becomes a practical, accessible, and sustainable option for diverse population groups and care contexts.

### 3.1 Implementation settings, target groups, and stakeholder engagement

Effective dissemination and implementation of the MDGs require a multisectoral approach that integrates diverse settings, target groups, and stakeholders.

Clinical environments remain a central entry point for embedding MD principles into healthcare practice. Here, the guidelines can be integrated into primary care and chronic disease management pathways, particularly for cardiovascular, metabolic, and oncological conditions. Healthcare professionals, including general practitioners, nurses, and dietitians, should be supported through structured training and practical tools to enable personalized, evidence-based dietary counseling (56). Integration into electronic health records, for example, through dietary assessment modules, adherence tracking, or automatic reminders, can support clinicians in delivering personalized, evidence-based dietary counseling. Beyond clinical practice, community and population-based initiatives are critical for achieving equitable and widespread adoption. Local health departments, schools, and community centers can serve as hubs for promoting the MD, emphasizing its health, cultural, and environmental benefits (24, 57). Educational settings play a pivotal role in shaping lifelong dietary behaviors. Integrating MDGs into school meal programs and nutrition curricula, complemented by family engagement and experiential learning such as cooking or gardening activities, can help instill healthy eating patterns from early life (58). While corporate wellness initiatives can promote adherence among adults through Mediterranean-style menus, educational workshops, and incentive-based challenges (59). Digital platforms and workplace settings also provide strategic opportunities for implementation. Web-based tools, mobile applications, and social media can extend the reach of MDGs through interactive resources such as meal planners, adherence tracking, and behavior-change prompts (60). Similarly, workplaces can promote MD-aligned habits through wellness programs, healthy cafeteria offerings, and incentive-based health initiatives (61).

The MDGs are designed to benefit the entire population, but their impact can be maximized through tailored approaches addressing the needs of specific groups. In clinical contexts, individuals with chronic diseases, such as cardiovascular disease, type 2 diabetes, cancer, and neurodegenerative disorders, derive particular benefit from adherence to the Mediterranean Diet, which supports both primary prevention and improved disease management. For the general adult population, the MD provides an evidence-based, culturally adaptable framework that promotes long-term health and reduces the risk of non-communicable diseases through its emphasis on plant-based foods, healthy fats, and traditional culinary practices (62). Children, adolescents, and pregnant women represent critical windows for early-life interventions. Promoting MD-based nutrition in schools and households fosters lifelong healthy behaviors and has been associated with improved maternal and child outcomes, including lower risks of gestational diabetes and low birth weight. Particular attention should be paid to children and adolescents, as the increasing prevalence of childhood overweight and obesity is closely linked to poor dietary patterns at early ages (63). Interventions targeting younger populations are vital to establish foundational healthy behaviors that persist in adulthood. Incorporating MD principles into school curricula, family routines, and institutional meal planning can serve as key levers for change during these formative years (64). Among older adults, adherence to MD patterns supports functional ability and quality of life, helping to mitigate frailty and sarcopenia. Finally, strategies must address vulnerable populations facing socioeconomic barriers to healthy food access. In these cases, adapting MDGs to reflect local food environments, supported by public policies, subsidies, and procurement programs, can enhance affordability and equity. Finally, the success of MDG implementation depends on the coordinated involvement of key stakeholders. Government ministries, including those of Health, Education, Agriculture, and Environment, should embed MDGs within broader national policies and ensure coherence with food procurement and fiscal measures. Public institutions, local health authorities, and research bodies provide structural backbone for applying and monitoring MDG-based interventions. Professional and scientific societies play an essential role in maintaining evidence-based practice through education and consensus-building among healthcare providers. Civil society organizations, patient groups, and consumer associations facilitate community engagement and adaptation of MDGs to local contexts, while the private sector contributes by reformulating products, promoting local and seasonal foods, and ensuring responsible marketing. Finally, traditional and digital media play a vital role in shaping public perception through targeted, culturally sensitive communication campaigns.

This integrated and tool-supported approach, linking settings, populations, and institutions, ensures that the MDGs are not only scientifically sound but also practically applicable, socially embedded, and scalable across healthcare, education, and policy systems.

### 3.2 Dissemination and implementation plan

The effective adoption of the MDGs necessitates a comprehensive and multilayered dissemination and implementation strategy that integrates both top-down institutional actions and bottom-up community engagement. Such an approach ensures that policy direction, service provision, and public understanding are aligned to create enabling environments for long-term dietary change (65). A foundational component of the implementation strategy involves education and training. Health professionals, educators, and social service workers must be equipped with up-to-date, evidence-based knowledge about the MDGs to promote consistent messaging and tailored guidance. This can be achieved through the development of dedicated training modules, continuing professional development programs, and flexible e-learning resources that address the scientific basis, practical application, and communication strategies related to the MD. Communication campaigns are essential to build public awareness and engagement. National and regional efforts should leverage both traditional media and digital platforms to disseminate messages that underscore the health benefits, cultural relevance, sustainability, and economic feasibility of the MD. Social marketing principles, community testimonials, and visual storytelling can foster resonance across diverse population segments and contribute to behavior change. Policy integration represents a critical axis of sustainability (66). The MDGs should be embedded within broader national strategies on food, nutrition, and non-communicable disease prevention, as well as sectoral guidelines for clinical care, school feeding programs, and institutional catering in settings such as hospitals and elder care facilities. Aligning MDGs with these frameworks facilitates institutional commitment and cross-sector coordination. Importantly, the MDGs should also be considered a central component within the Clinical Pathways (*Percorsi Diagnostico-Terapeutici Assistenziali, PDTA*), where they can serve not only as a therapeutic dietary approach but as a structured intervention strategy embedded in disease management protocols. Monitoring and evaluation are vital to assessing the effectiveness of implementation efforts. Standardized indicators and validated tools should be used to measure adherence to MD, track dietary outcomes, and evaluate health impacts (67). Validated instruments such as the Mediterranean Diet Adherence Screener (MEDAS) (31) or the KIDMED index (68) for younger populations, adapted to national contexts should be considered. The integration of these tools into national nutrition surveillance systems offer a ready-made infrastructure for this purpose, and their expansion to include MD-specific metrics should be encouraged. Quantitative indicators should track adherence scores, dietary diversity, and consumption of core food groups, while health-related outcomes such as anthropometric measures (e.g., Body mass index, waist circumference, etc.), lipid profiles, blood pressure, and NCD incidence can serve as proxy measures for long-term impact. Pilot and demonstration projects offer opportunities to test interventions in diverse geographic, cultural, and socioeconomic contexts. These projects can assess feasibility, acceptability, and cost-effectiveness, generating local evidence to inform scalable models. Successful initiatives can then serve as templates for broader rollout and policy refinement (69). Embedding monitoring and evaluation elements within existing national nutrition and health systems ensures that the MDGs remain dynamic and data-informed supporting continuous improvement and alignment with global public health objectives. Finally, the use of digital toolkits can enhance both professional practice and public engagement. Interactive web platforms, mobile apps, and user-friendly infographics can translate scientific recommendations into actionable, personalized guidance (70). These resources can support healthcare providers in counseling sessions, educators in classrooms, and individuals and families in their everyday food choices. Overall, the dissemination and implementation of the MDGs should be framed as an ongoing, adaptive process grounded in scientific evidence, stakeholder collaboration, and community empowerment. Such a dynamic approach can ensure that the MDGs become not only a dietary guideline but a lived, culturally relevant health-promoting practice. Feedback mechanisms linking healthcare professionals, policymakers, and communities are crucial for iterative refinement and responsive adaptation of interventions.

## 4 Discussion

The development of the MDGs within the framework of the Italian National Guidelines System (SNLG) represents a significant advancement in the field of nutritional policy and public health guidance. This initiative stands out not only for its methodological rigor, marked by the application of the GRADE and the NUTRIGRADE systems, but also for its emphasis on inclusivity, transparency, and contextual adaptability. Unlike many traditional dietary recommendations, which often lack standardization in development procedures (16), the MDGs were elaborated through a structured process involving a panel of experts and stakeholders across multiple sectors, supported by systematic reviews and economic analyses. Although the process reflects the rigor of systematic reviews, the MDGs were conceived as a guideline development project rather than a stand-alone review, and thus did not adopt the PRISMA flowchart format, which is specific to systematic review reporting. This comprehensive and evidence-based approach ensures that the MDGs are both scientifically robust evidence and aligned with Italy’s specific public health and clinical priorities. A key strength of the MDGs lies in their dual applicability: they serve as a preventive tool in the general population while also offering therapeutic benefits in clinical settings. The guidelines provide recommendations across multiple health domains, including cardiovascular, metabolic, oncological, neurological, and autoimmune diseases, at both primary and tertiary prevention levels. Finally, the MDGs address the diverse impacts of MD not only on health outcomes but also on economic sustainability and environmental preservation (25). Strong evidence from RTCs and observational studies supports the protective effect of MD adherence against major cardiovascular events (39) and type 2 diabetes (71). Similarly, moderate evidence suggests that MD can reduce the risk of certain cancers (43) and neurodegenerative diseases (48, 49), improve maternal-fetal outcomes (72, 73), and enhance quality of life in individuals with chronic or autoimmune conditions (51, 52). Hence, not all studies demonstrate consistent benefits of the MD across all health outcomes. Evidence related to oncological, neurocognitive, and autoimmune diseases remains heterogeneous, reflecting differences in study design, population characteristics, and adherence scoring systems. While the protective effects of the MD are strongly supported in cardiovascular and metabolic domains, results in other areas remain variable and warrant further investigation through high-quality, longitudinal, and interventional research. One of the most innovative aspects of MDGs is their redefinition of the MD. Moving beyond a narrow list of food components, the MD is conceptualized as a multidimensional lifestyle encompassing nutritional, sociocultural, environmental, and ethical elements (27). This updated definition incorporates the exclusion of alcohol consumption from its core recommendations, a significant departure from earlier interpretations that endorsed moderate wine intake (74). The decision to exclude alcohol from the definition of the MD reflects a growing scientific consensus on its health risks, even at low levels, and marks a shift toward a more protective dietary model (35). While total abstention is widely recognized as the healthiest option, particularly for younger populations, debate persists regarding the effects of low-to-moderate alcohol consumption, especially among male adults over 40. Traditional epidemiological studies suggest some protective effects on mortality, but these findings are increasingly challenged by Mendelian randomization studies (75) and recent Global Burden of Disease analyses, which find no safe level of alcohol consumption (76–78). Methodological limitations, such as confounding factors and misclassification, contribute to the conflicting results. Moreover, the perceived benefits of moderate drinking may reflect the generally healthier lifestyle of moderate drinkers rather than the alcohol itself (79). This confounding effect is particularly relevant to the interpretation of adherence scores used in epidemiological research on the MD. Several widely applied indices of MD adherence, such as the MDS or its derivatives, historically included moderate alcohol consumption, especially wine with meals, as a positive scoring component. Such inclusion may have inadvertently inflated the apparent health benefits of MD adherence by conflating the lifestyle factors of moderate drinkers with diet quality. In other words, part of the positive associations observed between MD adherence and health outcomes may reflect the broader lifestyle characteristics of moderate drinkers rather than the alcohol intake itself. Given alcohol’s addictive potential and associated health risks, abstention remains the most prudent public health recommendation. More definitive guidance will require evidence from well-designed RTCs, which are currently lacking (34, 36). Despite the considerable strengths of the MDGs, the process also encountered limitations that warrant critical reflection. One of the main challenges encountered was the heterogeneity in both the quality and type of evidence available across the various health outcomes, compounded by the variability in the adherence scoring systems used to define compliance with the MD. While cardiovascular and metabolic domains benefited from high-quality trials like PREDIMED (39), other areas, such as autoimmune disorders, musculoskeletal diseases, and mental health, relied primarily on observational studies with lower certainty (29). This disparity constrained the strength of certain recommendations and highlights the need for more RTCs and longitudinal research in underexplored domains. The strength of the evidence underpinning the MDGs is inevitably constrained by the heterogeneity of the included studies, the scarcity of randomized controlled trials, and the potential bias inherent in observational research, which may influence the consistency and generalizability of the findings. Another limitation concerns the operationalization and dissemination of the MDGs. Although the guidelines provide a robust foundation for dietary guidance, their real-world impact depends heavily on successful translation into policy and practice. Barriers such as socioeconomic inequalities, limited access to fresh and healthy foods, and varying levels of health literacy can undermine adherence, particularly among vulnerable groups (80). The guidelines acknowledge these barriers and propose tailored strategies to address them, including community engagement, targeted subsidies, and educational campaigns. However, the implementation of these strategies requires sustained political commitment, cross-sector collaboration, and adequate resource allocation (81). In terms of policy implications, the MDGs offer a replicable model for the development of dietary guidelines in other cultural and national contexts, particularly within the Mediterranean basin. Their integration into existing food and nutrition policies, health promotion strategies, and educational systems can help bridge the longstanding gap between clinical and population-level dietary recommendations. Furthermore, by demonstrating that high-quality guidelines can be developed within an institutional framework, the MDGs contribute to the international movement toward evidence-based nutrition policy and harmonization of dietary standards. The Italian MDGs extend their relevance beyond the national context, offering a replicable framework for countries seeking to develop culturally grounded yet evidence-based dietary guidance. Their methodological alignment with WHO frameworks for evidence-informed guideline development reinforces their international applicability. By integrating scientific rigor, cross-sector collaboration, and sustainability principles, the MDGs provide a transferable model adaptable to diverse healthcare and public health systems, demonstrating how traditional dietary patterns can be translated into scalable tools for addressing global nutrition transitions and non-communicable disease prevention.

The main strength of the MDG development process lies in the application of the SNLG framework, which ensures methodological rigor, transparency, and traceability through structured evidence appraisal systems such as GRADE and NUTRIGRADE. The inclusion of cost-effectiveness and sustainability dimensions represents a significant advancement, aligning the MDGs with global health agendas such as the WHO recommendations on sustainable healthy diets (82) and the UN Sustainable Development Goals (83), thereby enhancing their policy relevance. Nonetheless, challenges persist. Standardized approaches for evaluating the economic and environmental impacts of dietary patterns remain limited, and available data are often heterogeneous. Furthermore, the SNLG framework, originally designed for clinical interventions, required methodological adaptations to fit the complexity of dietary models, potentially affecting comparability and interpretation.

Despite these limitations, the integration of the SNLG approach within the MDGs represents a methodological innovation that bridges clinical and public health domains. It offers a scalable, harmonized model for guideline development across Mediterranean and EU countries, supporting the broader adoption of sustainable, evidence-based nutrition policies.

## 5 Conclusive remarks and future steps

**FIGURE 11:**
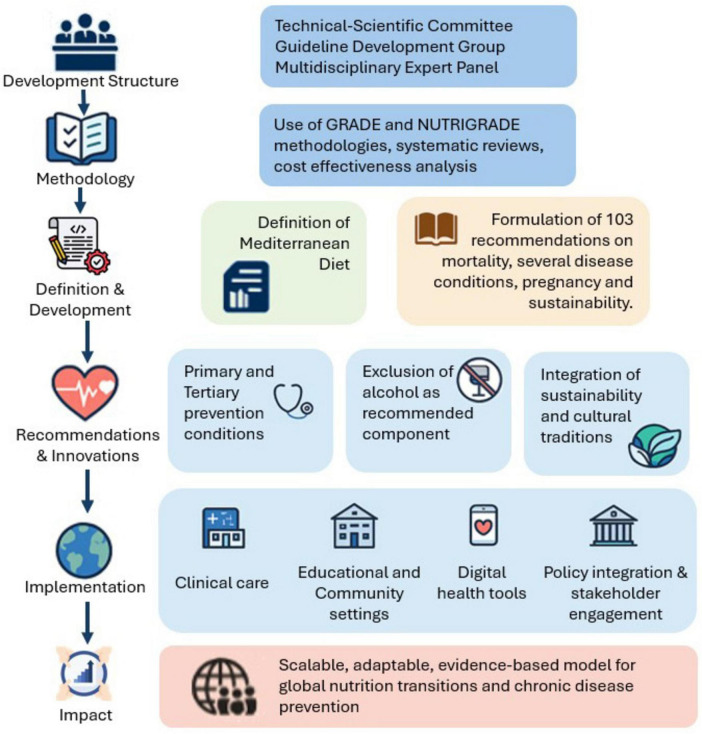
Mediterranean Diet Guidelines: development, evidence, and implementation.

In conclusion, the development MDGs summarized in Figure 11 demonstrates how a structured, evidence-based process can effectively translate nutritional science into public health recommendations. The synthesis of available evidence confirms strong protective effects of MD against cardiovascular and metabolic diseases, while highlighting more heterogeneous findings for oncological, neurocognitive, and autoimmune conditions. The methodology used ensured rigor and transparency, while identifying areas where additional high-quality research is needed. The MDGs mark a critical step forward in the evolution of nutritional recommendations. Their development shows how methodological rigor, stakeholder involvement, and cultural sensitivity can converge to produce guidelines that are not only scientifically sound but also contextually relevant and socially equitable. The integration of lifestyle factors, such as the importance of social and cultural eating practices (e.g., communal meals, the safeguarding of local food systems, prevention of foods waste, and the preservation of intergenerational culinary traditions), the exclusion of alcohol, and attention to food sustainability, enhances the relevance of these guidelines in modern dietary debate. Moreover, their attention to vulnerable groups, such as children, older adults, and pregnant women, reflects a life-course approach that aligns with contemporary public health priorities. Moving forward, continued investment in research, monitoring, and capacity-building will be essential to maximize the public health impact of the MDGs and support their implementation across diverse settings. As the burden of non-communicable diseases continues to rise, the MD, redefined and reinforced through the MDGs, offers a timely and transformative approach to promoting health, sustainability, and well-being. Investment in high-quality research, especially RTCs in underrepresented domains (e.g., mental health, autoimmune diseases, musculoskeletal conditions), will strengthen the evidence base and support the refinement of recommendations. In addition to that, the development of monitoring systems and validated tools to assess MD adherence and health outcomes will be vital for evaluating implementation progress. Lastly, promoting equity in dietary access and affordability, through economic measures, food policies, and public procurement strategies, must remain a priority to ensure that the benefits of the MD reach all segments of the population. Future updates of the MDGs should remain responsive to emerging evidence and evolving social contexts. By institutionalizing mechanisms for guideline revision, fostering international collaboration, and maintaining a participatory approach, the MDGs can serve as a dynamic and evolving reference model. In an era marked by complex health and environmental challenges, the MD, codified, redefined, and revitalized through these guidelines, continues to offer a scientifically grounded and culturally resonant path toward healthier people and a more sustainable environment.

## Footnotes

1
https://www.who.int/groups/guidelines-review-committee/about

2
https://www.iss.it/en/linee-guida1
